# Selective processing of clinical information related to correct and incorrect diagnoses: An eye‐tracking experiment

**DOI:** 10.1111/medu.15544

**Published:** 2024-09-24

**Authors:** Justine Staal, Jelmer Alsma, Jos Van der Geest, Sílvia Mamede, Els Jansen, Maarten A. Frens, Walter W. Van den Broek, Laura Zwaan

**Affiliations:** ^1^ Institute of Medical Education Research Rotterdam Erasmus University Medical Center Rotterdam Rotterdam The Netherlands; ^2^ Department of Internal Medicine Erasmus University Medical Center Rotterdam Rotterdam The Netherlands; ^3^ Department of Neuroscience Erasmus University Medical Center Rotterdam Rotterdam The Netherlands; ^4^ Department of Psychology, Education and Child Studies Erasmus School of Social and Behavioral Sciences Rotterdam The Netherlands; ^5^ Department of Emergency Medicine Erasmus University Medical Center Rotterdam Rotterdam The Netherlands

## Abstract

**Introduction:**

Diagnostic errors are often attributed to erroneous selection and interpretation of patients' clinical information, due to either cognitive biases or knowledge deficits. However, whether the selection or processing of clinical information differs between correct and incorrect diagnoses in written clinical cases remains unclear. We hypothesised that residents would spend more time processing clinical information that was relevant to their final diagnosis, regardless of whether their diagnosis was correct.

**Methods:**

In this within‐subjects eye‐tracking experiment, 19 internal or emergency medicine residents diagnosed 12 written cases. Half the cases contained a correct diagnostic suggestion and the others an incorrect suggestion. We measured how often (i.e. number of fixations) and how long (i.e. dwell time) residents attended to clinical information relevant for either suggestion. Additionally, we measured confidence and time to diagnose in each case.

**Results:**

Residents looked longer and more often at clinical information relevant for the correct diagnostic suggestion if they received an incorrect suggestion and were able to revise this suggestion to the correct diagnosis (dwell time: M: 6.3 seconds, SD: 5.1 seconds; compared to an average of 4 seconds in other conditions; number of fixations: M: 25 fixations, SD: 20; compared to an average of 16–17 fixations). Accordingly, time to diagnose was longer in cases with an incorrect diagnostic suggestion (M: 86 seconds, SD: 47 seconds; compared to an average of 70 seconds in other conditions). Confidence (range: 64%–67%) did not differ depending on residents' accuracy or the diagnostic suggestion.

**Discussion:**

Selectivity in information processing was not directly associated with an increase in diagnostic errors but rather seemed related to recognising and revising a biased suggestion in favour of the correct diagnosis. This could indicate an important role for case‐specific knowledge in avoiding biases and diagnostic errors. Future research should examine information processing for other types of clinical information.

## Abstract 2

Using eye movements, the authors reveal that use of specific types of clinical information does not differ between correct and incorrect diagnoses. Rather, case‐specific knowledge is key in avoiding diagnostic errors.

## 1 INTRODUCTION

Diagnostic errors, defined as missed, wrong or delayed diagnoses, are a large burden on patient safety. It is estimated most people will experience a diagnostic error during their lifetime, possibly with devastating consequences.^1^ Flaws in cognitive reasoning are thought to be among the main causes of diagnostic errors and are viewed as highly preventable.^2^, ^3^ However, given that reasoning processes occur rapidly and, in part, unconsciously, clinicians are not fully aware of the reasoning steps they take, nor of what errors they make or should correct.^4^, ^5^ It is therefore crucial to gain more insight into clinicians' reasoning processes during diagnosis via another method, such as measuring eye movements. Observed differences between correct and incorrect diagnoses could provide insights into the cognitive processes underlying diagnostic errors and following that, ways to prevent errors.

To understand how errors due to cognitive flaws can be prevented, it should first be understood how clinicians make a diagnosis. Clinical reasoning is mostly explained using the dual process theory,^6^ which distinguishes the fast and automated reasoning of System 1 and the more deliberate and conscious reasoning of System 2.^7^, ^8^, ^9^ The general explanation is that System 1 uses heuristics, or mental shortcuts, to quickly arrive at a solution. These heuristics are often efficient and useful^10^, ^11^, ^12^ but can also result in cognitive biases (systematic reasoning errors that occur when not all relevant information is considered^6^) and, subsequently, in diagnostic errors.^6^, ^13^, ^14^ The literature furthermore suggests that knowledge deficits could be a significant cause of errors.^15^, ^16^ What happens in the reasoning process when a diagnostic error occurs, compared to when a correct diagnosis is made, is still poorly understood. One possibility, however, is that inappropriate use of available clinical information could be an underlying factor in both instances, as both cognitive biases and knowledge deficits can result in the erroneous selection and interpretation of clinical information. Alternatively, erroneous selectivity could also be a cause of cognitive biases and knowledge deficits.

Previous research supports an association between erroneous information gathering or integration and diagnostic errors. Graber et al.^17^ found that faulty information synthesis was the most common cause of errors. Later studies analysing error cases similarly reported that errors such as not considering diagnoses or incorrectly weighing competing diagnoses occurred frequently.^18^, ^19^ Zwaan et al.^20^, ^21^ furthermore showed in record review studies that often, insufficient information was gathered and follow‐up on relevant findings was lacking. This selectivity was also associated with an increase in diagnostic errors and patient harm.^20^ Mamede et al.^22^ found that salient distracting features (i.e. pieces of information that grab attention because they are strongly associated to a certain diagnosis despite not being relevant to the correct diagnosis) caused diagnostic errors.

Several limitations should be kept in mind when interpreting previous research. First, previous studies often exclusively examined error cases and did not determine whether processes associated with errors also occurred in correctly diagnosed cases.^17^, ^18^, ^19^ This comparison is important to help us understand to what extent certain cognitive processes are part of the standard clinical reasoning process. Second, a majority of studies retrospectively assessed cases,^17^, ^18^, ^19^, ^20^, ^21^ a process that is susceptible to hindsight bias (i.e. when knowing the outcome of a case influences the judgement of the case^23^). Assessors might overestimate the likelihood that the diagnosis could have been made at that point in time.^24^ Prospective studies circumvent both issues, but to our knowledge, current prospective studies did not specifically examine selectivity in information processing in written case vignettes.

In the current study, information processing was measured using eye‐tracking,^25^ which allows an almost ‘live’ observation of clinicians' reasoning processes. Eye‐tracking data are interpreted as a measure of attention: information that receives more attention is processed more,^26^ and thus, a measure of attention is indirectly a measure of information processing.^27^ Residents diagnosed written clinical case vignettes while wearing a head mounted eye‐tracker. Each case contained either a most likely correct or a most likely incorrect diagnostic suggestion, meant to induce bias. We recognise it is not possible to arrive at the true correct diagnosis without further diagnostic work‐up; therefore, when we talk about a ‘correct’ or ‘incorrect’, this is based on the most likely diagnosis that the case was designed for. Previous studies have shown that diagnostic suggestions can influence the final diagnosis of clinicians of different levels of expertise.^28^, ^29^, ^30^, ^31^, ^32^, ^33^ This way, we could examine whether residents processed relevant information less when they arrived at an incorrect diagnosis. Information processing was measured as how long and how often residents looked at clinical information that was either relevant for the correct diagnostic suggestion or for the incorrect diagnostic suggestion. Additionally, we measured residents' diagnostic accuracy, their confidence in that diagnosis and total time spent to diagnose a case. We expected that residents would fixate longer and more often on clinical information that was relevant to their final diagnosis, regardless of the accuracy of the diagnostic suggestion. Furthermore, we expected that residents would be more confident if their diagnosis matched the diagnostic suggestion, regardless of their final accuracy. We expected no differences in the time spent on each case depending on the diagnostic suggestion or their accuracy.

## 2 METHODS

### 2.1 Design

The current study was a within‐subjects eye‐tracking experiment that aimed to prospectively investigate information processing during diagnosis in both error cases and correctly diagnosed cases. The study was approved by the medical ethical committee of the university (MEC‐2018‐1571). All participants gave informed consent. All methods were carried out in accordance with the relevant guidelines and regulations. Residents' eye movements were measured while they diagnosed 12 written clinical case vignettes with a diagnostic suggestion designed to induce bias. Each case contained a suggested provisional diagnosis, which was correct in six of the cases and incorrect in the other six. Half of the participants received a correct diagnostic suggestion for cases 1 to 6 and an incorrect diagnostic suggestion for cases 7 to 12. This was counterbalanced for the other half of the participants. The order of case presentation was randomised.

### 2.2 Participants

Nineteen internal medicine or emergency residents in their first to sixth year of training participated between November 2020 and August 2022 (Table 1). Residents were in training at the University Medical Center. They were recruited individually through mail and phone contact. Glasses or contact lenses were allowed if they did not distort the eye‐tracker signal.

**TABLE 1:** Participant demographics (internal medicine [n = 16] and emergency medicine residents [n = 3] taken together).

N	Age (SD)	Sex N (% female)	Years as resident (SD)
19	31 (3)	12 (63%)	3.2 (2)

### 2.3 Materials

#### 2.3.1 Cases

Twelve written clinical case vignettes were developed by one internist and independently diagnosed and confirmed by one emergency physician (Table 2). Cases concerned a variety of internal medicine and emergency medicine diagnoses that all junior doctors should be expected to recognise based on their teaching curriculum. Each case consisted of the history, medication details, physical examination findings and test results of fictional patients (Box 1). Cases were designed to have one correct diagnosis and one plausible, but incorrect, alternative suggestion. Clinical information in the cases included several distinguishing features that fit with either the correct or the incorrect diagnosis. When all considered together, these features weighed in favour of the correct diagnostic suggestion over the incorrect suggestion. It should be noted that final diagnostic accuracy was not the primary outcome of this study: Rather, we were interested in participants' the information processing underlying correct and incorrect diagnoses. The cases were piloted by third year residents and an emergency physician (N = 5) in three rounds.

**TABLE 2:** The average diagnostic accuracy for cases with the correct diagnostic suggestion (n = 10) and the incorrect diagnostic suggestion (n = 9), shown per case.

Case	Correct diagnostic suggestion	Diagnostic accuracy Mean (SD)	Incorrect suggestion	Diagnostic accuracy Mean (SD)
1	Ovarian torsion	0.30 (0.48)	Appendicitis	0.11 (0.33)
2	Nephrotic syndrome	0.50 (0.53)	Heart failure	0.33 (0.50)
3	Viral pericarditis	0.80 (0.42)	Pulmonary embolism	0.44 (0.53)
4	*Giardia lamblia* infection	0.70 (0.48)	Coeliac disease	0.44 (0.53)
5	Thrombotic thrombocytopenic purpura (TTP)	0.50 (0.53)	Immune thrombocytopenic purpura (ITP)	0.33 (0.50)
6	Sarcoidosis	0.40 (0.52)	Metastatic prostate cancer	0.11 (0.33)
7	Epstein–Barr virus (EBV) infection	0.88 (0.33)	Lymphoma	0.11 (0.33)
8	Toxic megacolon	1 (0)	Ileus	0.20 (0.42)
9	Hypoglycaemia	0.44 (0.53)	Benzodiazepine intoxication	0.11 (0.33)
10	Alcoholic hepatitis	0.11 (0.33)	Pancreatic cancer	0.11 (0.32)
11	Gout	0.44 (0.53)	Cellulitis	0 (0)
12	Obstructive sleep apnoea syndrome (OSAS)	0.66 (0.50)	Primary hyperaldosteronism	0.11 (0.35)

Box 1 **Example of a clinical case as presented during eye‐tracking (case 1). The case was presented in Dutch. The provisional diagnosis was either ovarian torsion or appendicitis (see Table 2).**

32‐year‐old woman presents to the emergency department with abdominal pain.

She has had the pain for 2 days.

The pain is sharp and radiates to the groin, currently rated 7/10.

It began around the umbilicus.

It is now moving to the right lower quadrant.

She is also experiencing nausea and has vomited twice.

During transportation, the pain increased with bumps on the road.

No fever was measured, but she feels clammy and sweaty.

She has a steady partner and is undergoing IVF treatment due to a desire to have children.

**Medical history**

Polycystic ovary syndrome.

Asthma.

Medication: none.

**Physical examination**

Moderately ill, painful woman.

Blood pressure 124/68; pulse 97; temperature 37.7.

Abdomen: sparse peristalsis.

Varied tympany.

Tenderness in the right lower quadrant.

Dubious rebound tenderness.

**Laboratory results**

Leukocytes 12 (reference 4–10).

CRP 39 (reference <10).

Urinalysis: leukocytes 2+, negative HCG.

No additional testing was performed.

**Provisional diagnosis:**

#### 2.3.2 Regions of interest

Regions of interest reflected the distinguishing features of each case and were defined a priori by one internist and one emergency physician, who independently marked the regions of interest and resolved discrepancies via discussion. These regions were classified based on whether the information they contained was relevant only to the correct diagnosis, or only to the incorrect diagnosis. All areas of a case that were not within a region of interest were designated as ‘background’ and were not analysed.

#### 2.3.3 Case presentation

The cases were scaled to fit a 1920 × 1080 px monitor. All information for one case was shown immediately on the same screen. All cases had a font size of 18 and double line spacing. No text was placed in the centre of the screen, to prevent accidental overlap between residents' gaze starting position and the regions of interest. All cases were written in Dutch.

#### 2.3.4 Eye‐tracker

The head‐mounted EyeLink II (SR‐Research, Canada) was used to record residents' eye movements during diagnosis. The EyeLink II tracks the corneal reflection and the pupil using infra‐red light at 500 Hz. We tracked the right eye, unless the resident indicated sight was worse in this eye. A 9‐point grid was used for calibration. Each calibration was validated a second time. The calibration was accepted if the inaccuracy between the gaze and the measurement was less than 4°. Residents were asked to keep their chin in a chin rest during the experiment, which kept their head stable and at approximately 60 cm distance from the monitor.

#### 2.3.5 Voice recorder

Residents were asked to state their most likely diagnosis out loud. This was recorded using a Basic voice recorder Premium. After transcription, the files were deleted.

#### 2.3.6 Survey

Additional outcome measures not directly related to the eye‐tracking measurement and feedback were presented via Qualtrics, an online survey tool.

### 2.4 Procedure

Residents received an information letter and signed informed consent. They were informed that the goal of the study was to investigate the cognitive processes underlying clinical reasoning. The experiment took approximately 20 to 30 minutes.

The eye‐tracker set‐up involved adjusting the head‐mounted eye‐tracker and seat to allow a comfortable position in the chin rest. The eye‐tracker was calibrated: Residents saw a black fixation cross in the centre of the screen before each case, to correct for possible drift in their position and to ensure that the gaze starting position did not overlap with the case text. The case was then shown. The provisional diagnosis was shown simultaneously with the case information. Residents were asked to diagnose 12 cases and indicate whether they agreed with the provisional diagnostic suggestion, which was presented as the suggested diagnosis from a colleague. If they did not agree, they were asked to provide their most likely diagnosis. There was no time limit for diagnosis and residents had to click after reading the case to indicate they wanted to provide a diagnosis. After clicking, a blank screen appeared and residents' most likely diagnosis was recorded using a voice recorder.

After all cases had been diagnosed, the eye‐tracker was removed and participants were asked to fill out a final set of questions in a Qualtrics survey. They were shown the history of each case again and were asked to indicate how confident they were in their diagnoses. They additionally provided demographic information. We performed a manipulation check by asking whether they suspected the goal of the study and provided the correct expert diagnosis for each case. Finally, they were debriefed about the goal of the experiment.

### 2.5 Outcome measures

The independent variable was the diagnostic suggestion: this suggestion was either the correct diagnosis or the incorrect, alternative diagnosis. The main dependent variables were diagnostic accuracy and the eye‐tracking measures reflecting information processing. Diagnostic accuracy was scored by one internist and one emergency physician, who independently assessed and assigned a score of 0 to incorrect diagnoses and of 1 to correct diagnoses. Discrepancies in scoring were resolved through discussion. The measures of information processing were dwell time (the time spent looking at a region of interest in milliseconds, converted to seconds) and the number of fixations on the regions of interest in each case. Additionally, residents' confidence (0: not confident to 10: very confident) and total time on task (in seconds) were measured.

Lastly, we asked residents to provide their medical specialty, age, sex and years of experience as a resident as demographic information.

### 2.6 Statistical analysis

First, bias induction was checked by comparing diagnostic accuracy in cases where a correct suggestion was provided with cases where an incorrect suggestion was provided, using a repeated measures *t*‐test.

The main analysis compared information processing in terms of the dwell time and number of fixations in regions of interest relevant for the correct diagnostic suggestion and regions of interest relevant for the incorrect diagnosis (the biased suggestion). Because the data consisted of within subjects observations with multiple measurements, we used linear mixed models for each dependent variable. In these models, the diagnostic suggestion and the final diagnostic accuracy were added as fixed factors. Furthermore, the interaction between these factors was analysed. For all models, participant was taken into account as a random factor, and case was accounted for as a covariate. For dwell time and number of fixations respectively, the total dwell time and total number of fixations were also taken into account as random factors. Post hoc analyses were conducted using pairwise comparisons adjusted using the least significant difference (LSD) method. All tests were performed in IBM SPSS Statistics for Windows (Version 26.0) and were considered significant at the α ≤ 0.05 level.

## 3 RESULTS

The 12 cases showed a considerable range in average diagnostic accuracy, varying from 8% to 63%. This was corrected for by using case as a covariate in the analysis.

For the linear mixed models, we included (N_*participants*_ = 19 multiplied by N_*cases*_ = 12) 228 data points. In 8 of the 12 cases, data from all participants could be used. Across these cases, six recordings were removed because the eye was not captured correctly.

### 3.1 Bias induction

Diagnostic accuracy was lower in cases with an incorrect suggestion (20%, 95% CI: 12%–27%) than in cases with a correct suggestion (57%, 95% CI: 48%–66%), *p* < 0.001, indicating that confirmation bias was successfully induced.

### 3.2 Manipulation check

None of the participants correctly guessed the true goal of the study.

### 3.3 Main analysis

The descriptive statistics of all outcome measures are shown in Tables 3 and 4.

**TABLE 3:** Participants' dwell time and number of fixations on regions of interest (ROI) relevant for the correct diagnostic suggestion and ROI relevant for the incorrect diagnostic suggestion.

Region of interest (ROI) Condition	Number of observations (N)	Dwell time (second) Mean (SD)	Number of fixations Mean (SD)
ROI Correct diagnostic suggestion			
Correct suggestion Correct diagnosis	64	4.0 (2.9)	16 (11)
Correct suggestion Incorrect diagnosis	48	3.9 (2.9)	16 (12)
Incorrect suggestion Correct diagnosis	22	6.3 (5.1)	25 (20)
Incorrect suggestion Incorrect diagnosis	88	3.9 (3.3)	17 (14)
ROI Incorrect diagnostic suggestion			
Correct suggestion Correct diagnosis	64	3.2 (3.0)	14 (12)
Correct suggestion Incorrect diagnosis	48	3.4 (2.9)	13 (11)
Incorrect suggestion Correct diagnosis	22	2.8 (3.4)	11 (14)
Incorrect suggestion Incorrect diagnosis	88	2.5 (2.3)	11 (10)

*Note*: Per ROI, averages are shown for each combination of diagnostic suggestion (correct or incorrect) and final diagnostic accuracy (correct or incorrect).

**TABLE 4:** Participants' average confidence and time to diagnose.

Condition	Number of observations (N)	Confidence (%) Mean (SD)	Total time (second) Mean (SD)
Correct suggestion Correct diagnosis	64	67 (14)	69 (33)
Correct suggestion Incorrect diagnosis	48	64 (18)	68 (36)
Incorrect suggestion Correct diagnosis	22	64 (17)	86 (47)
Incorrect suggestion Incorrect diagnosis	88	67 (19)	71 (39)

*Note*: Averages are shown for each combination of diagnostic suggestion (correct or incorrect) and final diagnostic accuracy (correct or incorrect).

### 3.4 Information processing

#### 3.4.1 Regions of interest relevant for the correct diagnostic suggestion

**FIGURE 1:**
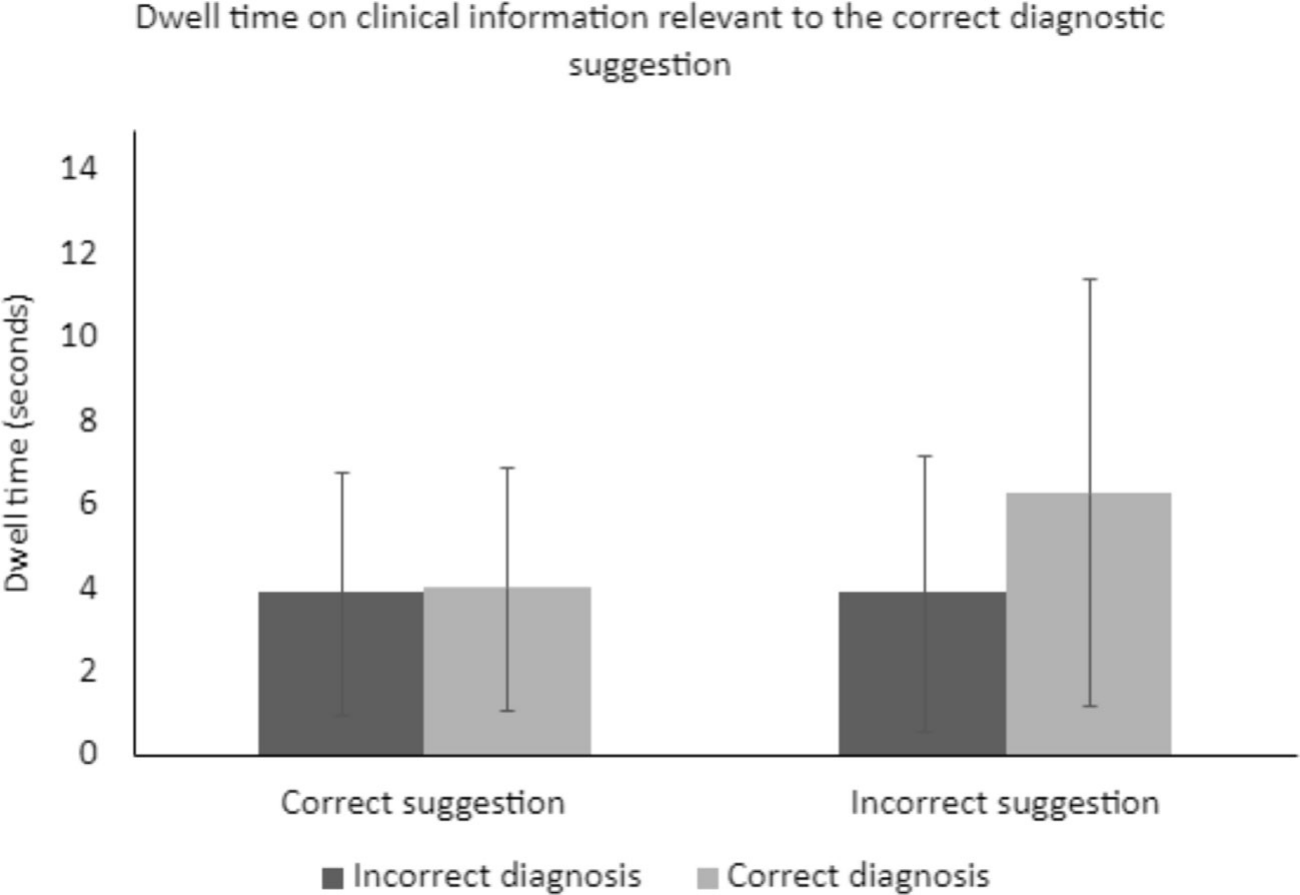
Interaction between the diagnostic suggestion and the final diagnosis for clinical information relevant to the correct diagnostic suggestion, on the average dwell time.

**FIGURE 2:**
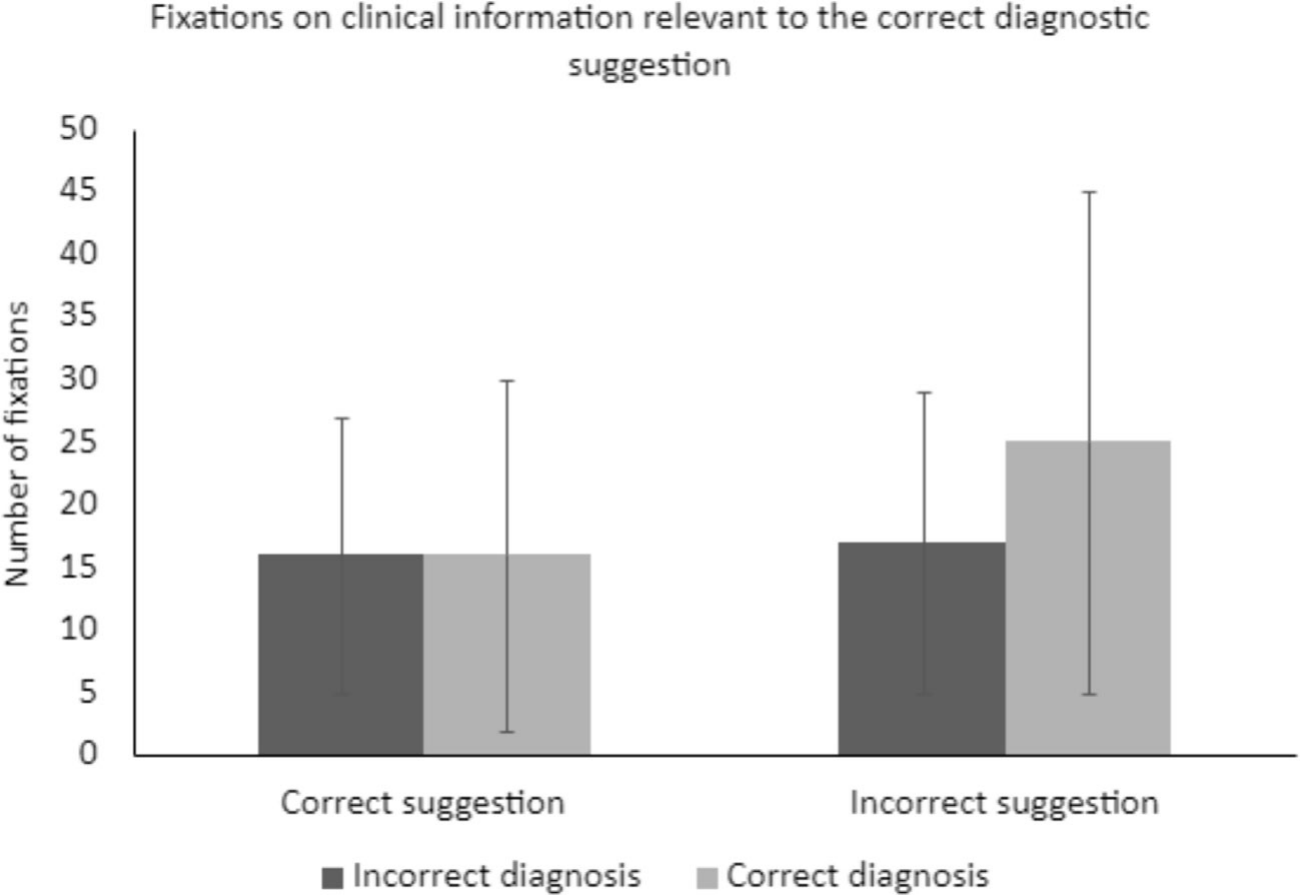
Interaction between the diagnostic suggestion and the final diagnosis for clinical information relevant to the correct diagnostic suggestion, on the average number of fixations.

Both dwell time, *F*(1, 216) = 5.30, *p* = 0.022, and the number of fixations, *F*(1, 220) = 5.69, *p* = 0.018, were higher for cases with an incorrect diagnostic suggestion than with a correct suggestion. Furthermore, dwell time was higher if participants diagnosed the case correctly, *F*(1, 218) = 5.2, *p* = 0.032. We observed a similar trend for the number of fixations, but this trend did not achieve significance, *F*(1, 220) = 3.71, *p* = 0.055. Finally, we observed an interaction between the diagnostic suggestion and diagnostic accuracy. Both dwell time, *F*(1, 219) = 7.97, *p* = 0.005 (Figure 1), and the number of fixations, *F*(1, 220) = 6.20, *p* = 0.014 (Figure 2), were higher if participants made a final correct diagnosis despite receiving an incorrect diagnostic suggestion, compared to when they did not correctly diagnose the case or when they received a correct diagnostic suggestion.

#### 3.4.2 Regions of interest relevant for the incorrect diagnosis

Both dwell time, *F*(1, 216) = 1.80, *p* = 0.181, and the number of fixations, *F*(1, 95) = 2.6, *p* = 0.110, did not differ between cases with an incorrect diagnostic suggestion compared to a correct suggestion. Similarly, both dwell time, *F*(1, 218) = 0.12, *p* = 0.915, and the number of fixations, *F*(1, 212) = 0.02, *p* = 0.896, were similar for correct and incorrect diagnoses. Furthermore, there was no interaction between the diagnostic suggestion and final diagnostic accuracy for either dwell time, *F*(1, 219) = 0.03, *p* = 0.874, or the number of fixations, *F*(1, 116) = 0.09, *p* = 0.761.

### 3.5 Confidence

Residents' confidence did not differ depending on the diagnostic suggestion, *F*(1, 218) = 0.03, *p* = 0.860, or their diagnostic accuracy, *F*(1, 219) < 0.01, *p* = 0.981. There was no interaction, *F*(1, 219) = 1.0, *p* = 0.319.

### 3.6 Time to diagnose

Residents spent more time on cases with an incorrect diagnostic suggestion compared to cases with a correct suggestion, *F*(1, 207) = 5.17, *p* = 0.024. Time to diagnose did not differ depending on diagnostic accuracy, *F*(1, 210) = 2.21, *p* = 00138. There was no interaction, *F*(1, 209) = 0.22, *p* = 0.639.

## 4 DISCUSSION

The current study aimed to examine residents' information processing in both error cases and correctly diagnosed cases. Information processing was defined as how long (dwell time) and how often (number of fixations) residents looked at clinical information that was either relevant for the correct diagnostic suggestion, or for the incorrect diagnostic suggestion. For the processing of information relevant for the correct diagnostic suggestion, we observed an increased dwell time and number of fixations in cases where residents received an incorrect diagnostic suggestion. Furthermore, dwell time was higher when residents gave a correct final diagnosis. This trend was similar for the number of fixations, but not significant. These main effects are further nuanced in the interaction we observed: specifically, dwell time and the number of fixations were increased in cases with an incorrect diagnostic suggestion, where the resident was able to revise this suggestion and give the correct final diagnosis (Figures 1 and 2). There were no differences in cases with an incorrect diagnostic suggestion and an incorrect final diagnosis, or cases with a correct diagnostic suggestion (regardless of the accuracy of the final diagnosis). No significant main effects or interactions were observed for the processing of clinical information that was relevant for the incorrect diagnostic suggestion. These findings were not in line with our hypothesis: we expected that residents would focus more on clinical information that was relevant for their own final diagnosis, regardless of whether this was the correct final diagnosis or not. Instead, we observed that information processing behaviours were similar between all combinations of diagnostic suggestion and final diagnostic accuracy, with the exception of when residents correctly revised an incorrect diagnostic suggestion.

Further, residents' confidence in their diagnosis did not differ depending on the diagnostic suggestion or their final diagnostic accuracy. We expected that confidence would increase if residents' final diagnosis was in line with the diagnostic suggestion they received; however, neither the accuracy of the diagnostic suggestion nor residents' final diagnostic accuracy seemed to influence their confidence. Finally, residents' time to diagnose was longer if they received a case with an incorrect diagnostic suggestion. This runs counter to our original hypothesis that time to diagnose would remain constant. However, the finding is in line with the observed interaction: residents spent more time processing information relevant to the correct diagnostic suggestion in cases with an incorrect diagnostic suggestion, and this also seems to have resulted in a longer overall time spent on the case.

Our findings suggest that selective information processing was not necessarily associated with diagnostic errors. If selective information processing was related to errors, we would have expected residents who gave a final incorrect diagnosis to focus more on clinical information relevant to the incorrect diagnostic suggestion—and ignoring clinical information relevant to the correct diagnostic suggestion. However, we did not observe differences in the processing of clinical information relevant to the incorrect diagnostic suggestion at all. Conversely, we found differences in processing only for clinical information relevant to the correct diagnostic suggestion. These differences were related to correctly revising an incorrect diagnostic suggestion and not to the occurrence of an incorrect final diagnosis. Thus, selective information processing seems important to revise potential erroneous diagnoses but does not seem associated with making an incorrect diagnosis. Our findings are in line with Kilian et al.^34^ who examined factors that led to the revision of a diagnostic error. They observed that participants were more likely to detect contradictory evidence if the diagnostic suggestion was incorrect and were more likely to attempt a revision. This is similar to our findings: Residents spent more time processing information in line with the correct diagnostic suggestion, that is, information contradictory with the incorrect suggestion. Contrary to our findings, Kilian et al.^34^ found that diagnostic accuracy did not improve, even if participants attempted a revision.

Furthermore, our observations can be linked to Mamede et al.'s^35^ finding that clinicians with higher knowledge (i.e. how many distinguishing disease features they recognised) were less susceptible to cognitive biases. Perhaps selectivity is guided by the prior knowledge to know what to look for. Similar findings are reported in eye‐tracking studies that examined clinical reasoning in visual diagnostic tasks. Generally, experts in the relevant clinical domain spent more time looking at areas in the diagnostic image that were clinically relevant^36^, ^37^, ^38^, ^39^ and were faster at identifying abnormalities in these areas compared to novices.^37^, ^40^ Selectivity in information processing, specifically on areas that might provide pertinent information, might therefore be an indication of one's ability to make the correct diagnosis.

The interaction between the incorrect diagnostic suggestion and the correct final diagnosis suggests that residents' information processing was not solely dictated by one's final diagnosis but also by a measure of effort expended, or awareness of the bias in the diagnostic suggestion. This finding may be in line with cognitive load theory, a framework concerned with how humans process information under the restraints of a limited working memory.^41^ Refuting an incorrect suggestion and starting from scratch to formulate a new diagnosis would introduce a higher load than simply agreeing with a correct suggestion, even if both diagnoses were ultimately correct.^42^ This might alternatively explain why selectivity was only observed when the suggestion was revised. Eye‐tracking measures are also an indication of the difficulty someone had with processing the presented information,^27^ and longer dwell time might indicate more effort. Revising a correct diagnostic suggestion with an incorrect final diagnosis, on the other hand, would likely be a similar process to refuting an incorrect suggestion, though this did not result in differences in selective information processing. Just detection of a bias or effort expended on a case can therefore not fully explain our findings, although these factors likely play a part in the measured eye‐tracking behaviours.

A question of causality does remain for the interaction we describe. Did residents who spent more time looking at relevant information arrive at the correct diagnosis because they processed this relevant information more? Or did residents with the appropriate knowledge to make the correct diagnosis know which information they needed to look for? Based on the previous studies in visual diagnosis,^36^, ^37^, ^38^, ^39^, ^40^ the latter explanation might be more likely: Experts, similar to those with higher knowledge, seem to know what information is clinically relevant and are able to make the correct diagnosis by focusing more on those areas. This could mean that residents who revised an incorrect diagnostic suggestion might have been more ‘expert’ on a certain diagnosis than their peers, which might have translated in the more ‘expert’ search pattern we observed. The differences that emerged in accuracy between cases might further indicate that case‐specific knowledge could play a role.

Some strengths and limitations of this study should be considered when interpreting the results. This study improves on previous studies into clinicians' reasoning processes by prospectively inducing diagnostic errors. The manipulation was successful, as we observed that participants who received an incorrect diagnostic suggestion also had a lower final diagnostic accuracy on these cases. Though the bias induction itself was successful, it is a reason of concern that diagnostic suggestions are so powerful, considering that suggestions are commonly provided in medical practice. Additionally, we assessed information processing in both correct and incorrect diagnoses. The use of eye‐tracking furthermore allowed a more precise observation of clinicians' reasoning than self‐report measures. We also included 12 observations per participant, and our participants were relatively experienced, which allowed us insight in the diagnostic reasoning process as it might occur in practice.

However, comparisons to practice were limited on other aspects. As this study was performed in a controlled laboratory setting, it could not take into account context‐specific influences on the diagnostic process. In recent years, clinical reasoning is recognised more and more as part of the complex healthcare context.^43^, ^44^ The findings from this laboratory study can be used to better understand the reasoning processes of individual clinicians, but future research should translate these findings to practice. Examples of context factors that could not be accounted for in this study include, the clinical case vignettes we used. The cases presented all necessary information simultaneously and the usual cyclicality of the diagnostic process and its progression over time were not incorporated. Residents could also not request extra information, so we could not measure whether follow‐up was neglected or whether residents would gather all relevant information themselves. This differs from, for example, dermatology and radiology settings, where the diagnoses rely much more on eyesight alone. We included both relevant information and additional, not strictly necessary information, that was not relevant for either the correct or the bias diagnosis so we could still observe whether residents were able to pick out the useful information when diagnosing a case. Furthermore, eye‐tracking experiments to assess information processing during diagnosis could be designed in numerous ways, for example by inducing a bias other than confirmation bias or by selecting different regions of interest, or cases of other levels of difficulty. Examining the effects of such factors could be valuable for future research. Finally, error rates in several cases were quite high. This could possibly be explained by the specialties of our participants: most participants were residents in internal medicine, but several were residents in emergency medicine. Our cases were a mix of diagnoses often seen in internal medicine and emergency medicine. It is possible that participants performed worse on diagnoses outside of their field of specialty, which might have impacted the diagnostic error rate.

In conclusion, selectivity in information processing likely plays a more complex role than simply being a direct cause of errors. The current results suggest that appropriate selectivity was associated with refuting incorrect diagnoses, whereas no specific differences were observed between correct and incorrect diagnoses in general. Selectivity in information processing might be a marker of cognitive processes underlying diagnostic errors rather than a cause of such errors. Eye‐tracking is a valuable method to more precisely test hypotheses regarding information processing and it could be useful in refuting or confirming assumptions about information processing that could distinguish between the role of knowledge and biases in reasoning. Future research should include more participants to assess individual differences and should explore the influence of many more factors, such as different types of information or cognitive biases on clinicians' information processing. Additionally, the role of prior knowledge or experience in identifying information relevant or irrelevant to the diagnosis of a case should be explored. This knowledge could guide, for example, teaching of critical knowledge in diagnostic reasoning. Better understanding how information processing occurs and what assumptions can be made about information processing during diagnosis will require more research.

## AUTHOR CONTRIBUTIONS

All authors had full access to all the study data and take responsibility for the integrity of the data and the accuracy of the analysis.

## CONFLICT OF INTEREST STATEMENT

The authors declare that they have no competing interests.

## ETHICS STATEMENT

The study was approved by the medical ethical committee of the University Medical Center (MEC‐2018‐1571). All participants gave informed consent. All methods were carried out in accordance with the relevant guidelines and regulations.

## Data Availability

The datasets used and/or analysed during the current study available from the corresponding author on reasonable request.
